# Critical role of gap junction communication, calcium and nitric oxide signaling in bystander responses to focal photodynamic injury

**DOI:** 10.18632/oncotarget.3553

**Published:** 2015-03-12

**Authors:** Bianca Calì, Stefano Ceolin, Federico Ceriani, Mario Bortolozzi, Andrielly H.R. Agnellini, Veronica Zorzi, Andrea Predonzani, Vincenzo Bronte, Barbara Molon, Fabio Mammano

**Affiliations:** ^1^ Foundation for Advanced Biomedical Research, Venetian Institute of Molecular Medicine, Padua, Italy; ^2^ University of Padua, Department of Surgery Oncology and Gastroenterology, Oncology and Immunology Section, Padua, Italy; ^3^ University of Padua, Department of Physics and Astronomy, Padua, Italy; ^4^ IRCCS, Istituto Oncologico Veneto, Padua, Italy; ^5^ Verona University Hospital, Department of Pathology and Diagnostics, Immunology Section, Verona, Italy; ^6^ Present address: CNR, Institute of Cell Biology and Neurobiology, Monterotondo (RM), Italy

**Keywords:** cancer, photodynamic therapy, nitric oxide, calcium signaling, connexins

## Abstract

Ionizing and nonionizing radiation affect not only directly targeted cells but also surrounding “bystander” cells. The underlying mechanisms and therapeutic role of bystander responses remain incompletely defined. Here we show that photosentizer activation in a single cell triggers apoptosis in bystander cancer cells, which are electrically coupled by gap junction channels and support the propagation of a Ca^2+^ wave initiated in the irradiated cell. The latter also acts as source of nitric oxide (NO) that diffuses to bystander cells, in which NO levels are further increased by a mechanism compatible with Ca^2+^-dependent enzymatic production. We detected similar signals in tumors grown in dorsal skinfold chambers applied to live mice. Pharmacological blockade of connexin channels significantly reduced the extent of apoptosis in bystander cells, consistent with a critical role played by intercellular communication, Ca^2+^ and NO in the bystander effects triggered by photodynamic therapy.

## INTRODUCTION

The phrase “bystander effects” was initially adopted in a radiotherapy context to account for responses observed in cellular systems that have not been directly traversed by ionizing radiations but are in close proximity to irradiated cells [1, 2]. Bystander effects triggered by ionizing radiations in tumor and tumor-infiltrating cells include altered gene expression, DNA damage, mutation, malignant transformation and cell death [3-9]. Bystander responses have been observed also as a consequence of other insults including ultraviolet radiation, heat, chemotherapy agents and photodynamic therapy; however the underlying mechanism and role in clinically relevant scenarios remain incompletely defined [1, 2].

Photodynamic therapy is a photochemistry-based approach, adopted primarily in oncology, ophthalmology and dermatology, which uses a light-sensitive chemical, termed photosensitizer, and light of appropriate wavelengths to impart cytotoxicity by generation of singlet oxygen [10] and other reactive molecular species [11]. Multiple signaling cascades and sub−cellular organelles are concomitantly affected in cells exposed to photodynamic stress, including adenylate cyclase, receptor tyrosine kinases, MAP kinases, phosphatidylinositol 3-kinase, various protein kinases and phosphatases, transcription factors, ceramide, the plasma membrane, mitochondria and the endoplasmic reticulum (ER) [12-16].

Nitric oxide (NO) has long been implicated in the apoptotic processes triggered by photodynamic therapy [17-19]. Nanomolar concentrations of NO reversibly inhibit cytochrome c oxidation and mitochondrial respiration; higher concentrations can irreversibly inhibit the respiratory chain [20], leading to the opening of the permeability transition pore and cell death [21, 22]. Its large diffusion coefficient (*D*_NO_ = 3300 μm^2^/s) [23] makes NO a prime candidate mediator of bystander responses. In addition, photodynamic stress has been causally associated with disruption of Ca^2+^ homeostasis and endoplasmic reticulum (ER) depletion [16, 24-26]. NO production by nitric oxide synthases (NOS) is controlled by enzyme binding to calmodulin (CaM) [27]; full activation of target proteins by CaM typically requires occupancy of its four Ca^2+^−binding sites [28].

Here we used C26GM mouse colon carcinoma cells [29] as a model system to explore bystander effects and the interplay between NO and Ca^2+^ signaling in the context of photodynamic therapy.

## RESULTS

We cultured C26GM cells [29] under standard conditions and co-loaded them with the commercially available and well-characterized photosensitizer AlClPc [30-35], the ratiometric fluorescent Ca^2+^ reporter fura-2 [36] and the selective turn-on fluorescent NO reporter CuFl [37, 38]. We photo−activated AlClPc for 60 s within a 5 μm diameter (Ø) area of a single cell in the culture at an irradiance of 60 μW/μm^2^, using a 671 nm diode-pumped solid-state laser connected to a fluorescence microscope. These stimulation conditions, which we refer to as *focal photodynamic injury*, were maintained for all experiments shown in this article.

**Figure 1:**
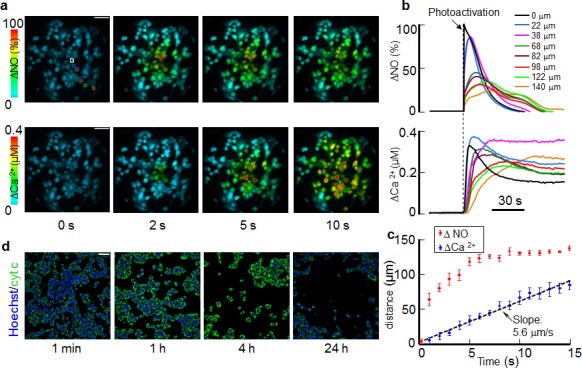
Focal photodynamic injury, i.e photo−activation of the photosensitizer AlClPc for 60 s in a single cell of a C26GM mouse colon carcinoma cell culture, triggers NO and Ca^2+^ signals that depart from the irradiated cell and rapidly invade bystander cells; these events are followed by cytochrome c release and widespread cell death. (a) Representative false−color images of simultaneously recorded cytosolic NO (top) and Ca^2+^ (bottom) concentration changes (Δ) during focal photodynamic injury; the irradiated cell is encased in a white region of interest (ROI); scale bar, 50 μm. (b) Single-cell fluorescence traces obtained as pixel averages from the corresponding (color-matched) ROIs in (a); irradiated cell responses are shown as black traces; the vertical dashed line marks the onset of laser irradiation; ΔNO data were normalized to the corresponding maximal response in the irradiated cell (see Methods); (c) The distance at which bystander cell signals reach 50% of their first peak amplitude is shown as a function of time after the onset of focal photodynamic injury. Data are mean ± s.e.m. from *n* = 6 cultures; the dashed line is a least square linear fit with a slope of 5.6 μm/s. (d) Cultures were rapidly fixed at shown time points after focal photodynamic injury and immunostained with a cytochrome c antibody and the nuclear counter stain Hoechst; note that images in (d) are from different cultures, whereas those in (a) are all from the same culture; scale bar, 25 μm.

By capturing CuFl and fura-2 fluorescence images in rapid sequence, we determined that AlClPc photo−activation reliably caused elevation (Δ) of cytosolic NO and Ca^2+^ levels in the irradiated cell. Within seconds, ΔNO and ΔCa^2+^ signals were detected in all (bystander) cells in the ~300 μm Ø field of view (Figure 1a-c). We obtained similar results in C26GM tumors grown within a dorsal skinfold chamber [39] implanted on BALB/c mice (Supplementary Figure 1), suggesting that the underlying signaling mechanisms are relevant for *in vivo* photodynamic therapy. We also tested focal photodynamic injury protocol in a different tumor cell line (fibrosarcoma, MCA-203) *in vitro*, and we obtained qualitatively similar results both for ΔNO and ΔCa^2+^ signals (Supplementary Figure 2). Immunostaining at different time points after focal photodynamic injury revealed cytochrome c release and cell loss progressing radially from the irradiated cell. The process ensued in near-complete depopulation of the field of view within 24 h following focal photodynamic injury (Figure 1d). ΔCa^2+^, ΔNO and cytochrome c signals were never detected during or after laser irradiation at 671 nm if AlClPc was omitted from the loading solution (3 out of 3 cultures).

**Figure 2:**
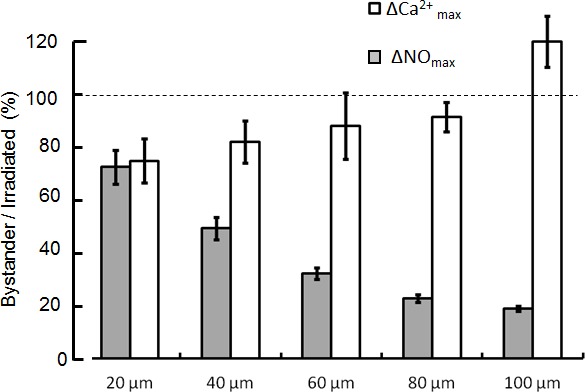
Maximal increments of NO (ΔNO) and Ca (ΔCa) levels evoked by focal photodynamic injury in bystander cells as a function of distance from the irradiated cell Data are mean ± s.e.m. from n = 3 cultures and were normalized to the corresponding maximal response in the irradiated cell.

Accurate temporal and spatial analysis of CuFl fluorescence emission (see Methods) highlighted strikingly different kinetics of ΔNO and Ca^2+^ signals. In the irradiated cell, ΔNO raised to 90% of its maximum value, ΔNO_max_, in < 300 ms, whereas the maximum ΔCa^2+^ increment, ΔCa^2+^_max_, occurred only 9.8 ± 1.0 s after the onset of photostimulation (mean ± s.e.m., *n* = 6 cultures). In bystander cells, (i) ΔNO peaked once or more depending on the distance from the site of irradiation and returned to baseline within 90 s in all cells; (ii) Ca^2+^ signals were progressively delayed at increasing distance from the irradiated cell, corresponding to the cell-to-cell propagation of a radial wave proceeding from the irradiated cell and travelling through the bystander cell population with average speed of 5.6 ± 1.1 μm/s (mean ± s.e.m., *n* = 6 cultures; Figure 1c). As shown in Figure 2, ΔNO_max_ decreased rapidly within ~60 μm from the irradiated cell, but less rapidly outside this range. By contrast, ΔCa^2+^_max_ showed a clear tendency to increase at increasing distance from the photo−activation site. At the periphery of the field of view, bystander ΔCa^2+^_max_ exceed the ΔCa^2+^_max_ of the irradiated cell by ~20%, on average.

**Figure 3:**
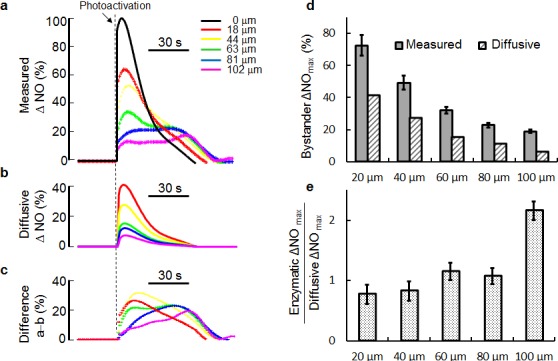
Comparison of experimental and model responses highlights dual contribution to NO signaling in bystander cells (a) Experimental ΔNO traces evoked by focal photodynamic injury at increasing distances from the irradiated cell (black solid line). (b) ΔNO signals in bystander cells predicted by a purely diffusive model using the irradiated cell signal in (a) as input and a diffusion coefficient *D*_NO_ = 3300 μm^2^/s. (c) Differences between the traces shown in (a) and (b), which we interpret as enzymatic contributions to bystander responses. (d) Maximal measured and diffusive NO level increments (ΔNO_max_) in bystander cells vs. distance from the irradiated cell. (e) Ratio of enzymatic ΔNO_max_ over diffusive ΔNO_max_ vs. distance from the irradiated cell. Measured data in (d) and (e) are mean ± s.e.m. from *n* = 3 cultures; those in (d) were normalized to the corresponding maximal response in the irradiated cell.

To get deeper insight into the intracellular and intercellular dynamics of ΔNO signals evoked by focal photodynamic injury, we created a mathematical model (see Methods, Equation 2, Supplementary Methods and Supplementary Figure 3) assuming that NO: (i) is generated within and released from the irradiated cell; (ii) diffuses freely across the extracellular space; (iii) passes freely through cell membranes of bystander cells, in which it is finally detected by pre−loaded CuFl. We used one of the ΔNO traces measured in an irradiated cell as input to this model and computed ΔNO bystander responses. The results of this analysis (Figure 3) show that ΔNO responses measured in bystander cells (Figure 3a) largely exceed those predicted based solely on NO diffusion (Figure 3b). The differences between measured and diffusive ΔNO signals provide estimates of the alternative generation of NO in bystander cells, likely by its enzymatic production by NOS (Figure 3c). Both the measured NO level increments and the purely diffusive component (estimated by the mathematical model) are monotonically decreasing functions of distance from the irradiated cell (Figure 3d), however the diffusive contribution exhibits a faster spatial rate of decrease. Consequently the ratio of measured minus diffusive (i.e. enzymatic) ΔNO_max_ over diffusive ΔNO_max_ shows a tendency to increase towards the periphery of the field of view, where it is >2 (Figure 3e).

Altogether, the results presented in Figures 1, 2, 3 suggest that (i) NO is generated almost immediately within the irradiated cell upon AlClPc photo−activation, (ii) diffuses rapidly to bystander cells where (iii) its levels are further increased by a Ca^2+^−dependent enzymatic production driven by the underlying Ca^2+^ wave.

**Figure 4:**
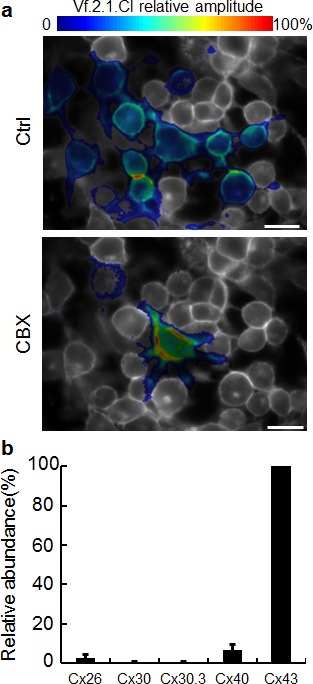
C26GM cells are coupled by gap junction channels (a) Coupling assay based on voltage imaging with the Vf.2.1.Cl membrane potential sensor shows cells are coupled by gap-junction channels (Ctrl), which can be blocked by carbenoxolone (CBX, 100 μM); scale bar, 50 μm. (b) Relative abundance of connexin transcripts in C26GM cultured cells assayed by qPCR.

To test these hypotheses we performed a series of pharmacological interference experiments. We noted that the relatively low value of the Ca^2+^ wave speed is compatible with a propagation mechanism whereby diffusion of soluble messengers, such as IP_3_, through gap junction channels plays a significant role [40]. Gap junction communication has been repeatedly implicated in bystander responses to ionizing radiation [41-46]. Therefore, we assayed C26GM cultures for the presence of functional intercellular channels using a novel, highly sensitive approach [47], based on a combination of patch clamp and voltage imaging with the membrane potential reporter Vf.2.1.Cl [48]. Our results (Figure 4a) indicate that cultured C26GM cells form functional syncytia since (i) electrical signals delivered to the patch-clamped cell invaded a number of other cells in the culture and (ii) electrical coupling was reversibly abrogated by carbenoxolone (CBX), a widely used non-specific inhibitor of connexin-made channels [49]. qPCR analysis for five different connexins expressed in various tumors [50-52] singled out Cx43 as the predominant isoform expressed by C26GM cells, whereas Cx40 and Cx26 provide minor contributions (Figure 4b).

**Figure 5:**
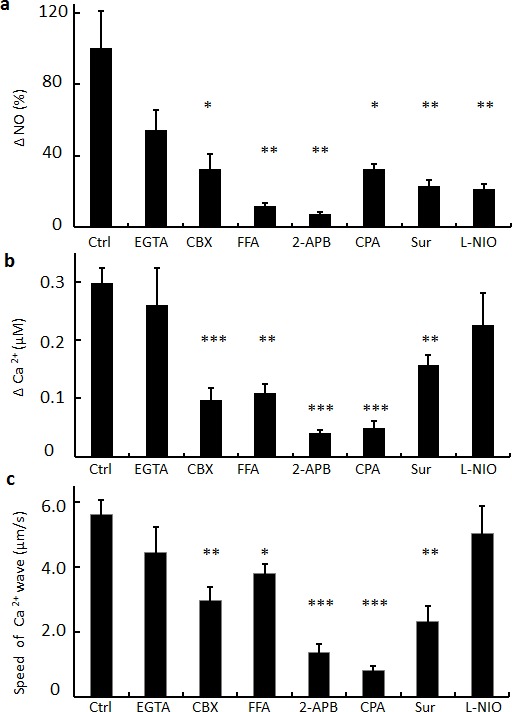
Summary of pharmacological interference experiments Cells were incubated for 15−20 min with shown drugs prior to focal photodynamic injury; concentrations: EGTA, 100 μM (in nominally Ca^2+^−free medium); CBX, 100μM; FFA, 100 μM; 2-APB, 100 μM; CPA, 30 μM; suramin (Sur), 200 μM; L-NIO, 10 μM. Data in (a−b) are mean ± s.e.m. of signals measured in 36 randomly selected bystander cells located in an annular region at the periphery of the field of view (between 75 μm and 120 μm from the irradiated cell). (a) NO level change (ΔNO) normalized to the average change measured in control conditions (Ctrl). (b) Cytosolic Ca^2+^ concentration change (ΔCa^2+^). (c) Speed of Ca^2+^ wave elicited by focal photodynamic injury.

Figure 5 illustrates the effects of gap junction inhibitors and other drugs on the speed of the intercellular Ca^2+^ wave, the ΔCa^2+^ and ΔNO signals evoked in bystander cells by focal photodynamic injury. Both CBX and flufenamic acid (FFA), another commonly used, non-specific inhibitor of connexin-made channels [49], caused a significant reductions of these three parameters. We also examined the consequences of perturbing Ca^2+^ homeostasis prior to focal photodynamic injury. The mild inhibition we observed in Ca^2+^−free extracellular medium (EGTA) implies negligible contribution of Ca^2+^ entry to bystander responses. Conversely, all three parameters were significantly reduced if ER Ca^2+^ levels were lowered by incubating C26GM cultures with cyclopiazonic acid [53] (CPA), a specific inhibitor of sarco/endoplasmic reticulum Ca^2+^−ATPase (SERCA pumps), in Ca^2+^ free medium. The most pronounced reductions of ΔNO and Ca^2+^ bystander signals were obtained with 2APB, a non-specific inhibitor of IP_3_ receptors (IP_3_R) [54]. ΔNO and ΔCa^2+^ signals and the speed of the intercellular Ca^2+^ wave were significantly attenuated also by suramin [55], suggesting that paracrine signaling mediated by ATP may play a role. Finally, the potent irreversible NOS inhibitor N-iminoethyl-L-ornithine (L-NIO) [56] significantly reduced bystander NO responses (Figure 5a) without affecting bystander Ca^2+^ signaling (Figure 5b, C).

**Figure 6:**
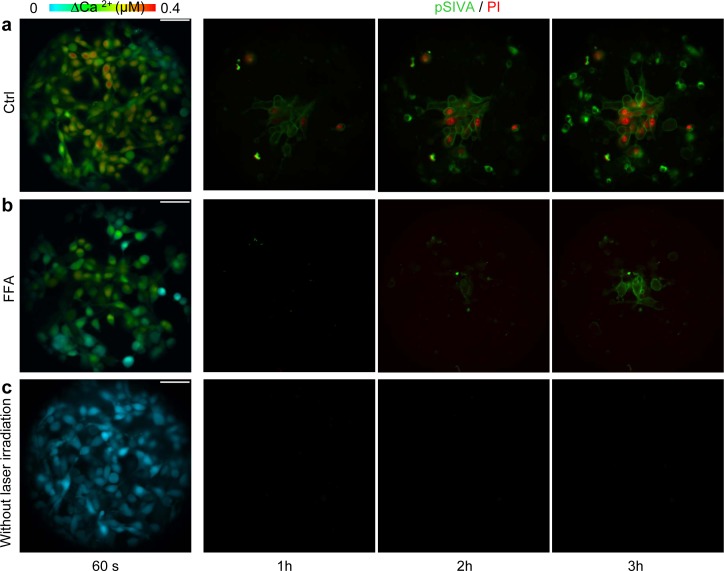
Apoptosis assays The leftmost panels in (a−c) are representative false−color images of cytosolic Ca^2+^ concentration changes (ΔCa^2+^) obtained as maximal projection rendering of all frames recorded while imaging C26GM cell cultures for 60 s. The same cultures were then immediately used for time-lapse microscopy, applying the polarity sensitive probe for the spatio-temporal analysis of apoptosis pSIVA-IANBD (pSiva, green) together with propidium iodide (PI, red) as instructed by the manufacturer. In (a) and (b), focal photodynamic injury was performed as usual. In (c), cells were loaded with AlClPc as in (a) and (b), but the laser was not activated. In (b), FFA was maintained throughout the recording. Scale bars, 50 μm.

**Figure 7:**
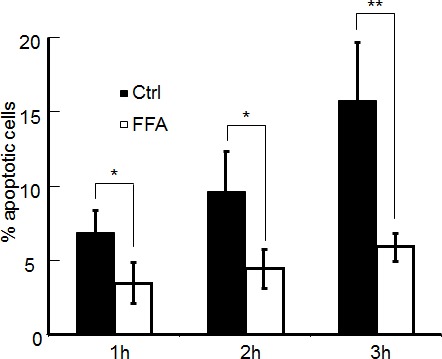
Percentage of apoptotic cells at three time points following focal photodynamic injury in control conditions and in the presence of FFA Data are mean ± s.e.m. from *n* = 3 cultures in each condition.

The results presented so far support the notion that coupling through gap junctions significantly contributes to the ΔCa^2+^ and ΔNO signals evoked by focal photodynamic injury, by permitting cell-to-cell propagation of ER− and IP_3_R−related Ca^2+^ signals through the network of bystander cells. To evaluate the relevance of gap junction communication also for the apoptotic effects triggered by focal photodynamic injury, we performed additional experiments in C26GM cultures co-loaded with AlClPc and fura-2. At the end of laser irradiation, we switched from Ca^2+^ imaging to a time-lapse protocol based on staining with propidium iodide and pSIVA-IANBD (Figure 6), an annexin-based polarity sensitive probe for the spatiotemporal or kinetic analysis of apoptosis [57]. Under control conditions, the irradiated cell exhibited detectable pSIVA-IANBD signals as soon as 30 min after focal photodynamic injury. Both pSIVA-IANBD and propidium iodide signals became detectable in the irradiated cells and the nearest neighbours within 1 h, and reached the limits of the field of view within 3 h (Figure 6a). These processes were greatly attenuated and slowed down by the gap junction blocker FFA (Figure 6b). No toxicity was observed in C26GM cultures loaded with AlClPc but not exposed to laser irradiation at 671 nm (Figure 6c). The fraction of C26GM cells exhibiting apoptotic signals as a consequence of focal photodynamic injury increased almost linearly with time, exceeding 15% of the population within 3h; this apoptotic rate was significantly reduced, by a factor >2.5, in the presence of FFA (Figure 7).

## DISCUSSION

The overall goal of this study was to investigate the interplay between bystander NO and Ca^2+^ signaling and the role played by gap junction communication in photodynamic therapy. A striking outcome of our work is that photoactivation in a single cell of a well known photosensitizer, AlClPc [30-35], a condition we refer to as focal photodynamic injury, results in cytochrome-c release and apoptosis, which progress radially from the irradiated cell and cause massive cell demise in the ~300 μm Ø field of view within 24 h (Figures 1, 6 and 7). The similarity between the responses evoked by focal photodynamic injury *in vitro* (Figure 1) and *in vivo* (Supplementary Figure 1) suggests that these finding may be relevant for photodynamic therapy treatments.

By performing fluorescence imaging with CuFl, a probe that reacts rapidly and specifically with NO over other potentially interfering reactive molecules [37, 38], we showed here that the irradiated cell acts as a powerful source of NO, which is generated almost immediately upon photo−activation of the photosensitizer and rapidly diffuses to bystander cells. To account for the extremely rapid rise in NO levels during AlClPc photo−activation, we hypothesize that NO is buffered by this phthalocyanine photosensitizer and released from the cell exposed to laser irradiation. This mechanism is supported by quantum chemistry computations, showing that the metal center of AlClPc binds NO in a redox-dependent manner [58]. Indeed, there is ample evidence that phthalocyanines can be specifically modified to act as highly efficient NO reservoirs and photosensitive NO donors that also produce singlet oxygen [59, 60].

Photoactivation of AlClPc disrupts Ca^2+^ homeostasis in the irradiated cell and initiates a radial Ca^2+^ wave. Reactive oxygen and nitrogen species (ROS/RNS) influence Ca^2+^ homeostasis via inhibition of PMCA and SERCA pumps and/or by increasing activity of ER release channels (both IP_3_Rs and ryanodine receptors) [61, 62]. Current hypotheses propose an alteration of PMCA Tyr589, Met622 and Met831 residues whereas SERCA activity has been shown to be inhibited by ROS/RNS modification of cysteine (and tyrosine) residues [61].

The original analysis method we developed for CuFl signals (see Methods, Equation 1 and Supplementary Methods) has been instrumental to demonstrate that NO levels increase faster than Ca^2+^ levels at all locations. Comparison of measured ΔNO signals to those predicted by a simple diffusive model indicates that NO diffusion from the irradiated cell accounts only for a fraction of the responses detected by CuFl in bystander cells (Figure 3). We have equated (Ca^2+^ dependent) enzymatic NO production in bystander cells (Figure 3c) to the difference between measured (Figure 3a) and diffusive ΔNO signals (Figure 3b). ΔNO responses predicted by the diffusive model were computed assuming that NO influx into bystander cells is not hindered by the plasma membrane. However, the exchange of NO between extracellular medium and cytoplasm was recently proposed to require connexin hemichannels [63]. If the plasma membrane indeed slows down NO influx, bystander responses in Figure 3b are over-estimated and, correspondingly, those in Figure 3c are under-estimated. Based on our data, the latter accounts for (at least) ~50% to ~70% of ΔNO signals in bystander cells. Furthermore, the relative importance of enzymatic production versus NO diffusion from the irradiated cell increases with increasing distance from enzymatic NO production (Figure 3d, e). This conclusion is supported by the >70% reduction imparted by the NOS inhibitor L-NIO [56] to the ΔNO responses of bystander cell at the periphery of the filed of view (Figure 5a).

Altogether, the results of our modeling efforts and pharmacological interference experiments (Figures 3, 5, 6, 7 and Supplementary Figure 3) are consistent with a scheme in which NO produced in the irradiated cell diffuses rapidly to bystander cells, where its levels are further increased by a slower mechanism compatible with Ca^2+^−dependent enzymatic production driven by the underlying Ca^2+^ wave, which initiates in the irradiated cell and propagates to bystander cells at constant speed (Figure 1c).

In accord with our proposed hypothesis, we have unequivocally showed that C26GM cells are coupled by gap junction channels mainly formed by Cx43 subunits (Figure 4). Furthermore, pharmacological perturbation of either gap junction communication or intracellular Ca^2+^ homeostasis reduced significantly the speed of the intercellular Ca^2+^ wave as well as ΔCa^2+^ and ΔNO bystander responses, whereas NOS inhibition by L-NIO [56] significantly reduced ΔNO responses without affecting appreciably Ca^2+^ signaling. Of notice, ΔCa^2+^_max_ in bystander cells shows a tendency to increase with distance from the irradiated cell (Figure 2), implying an active, self-regenerative mechanism [40]. In this regard, the sensitivity of Ca^2+^ wave speed, ΔCa^2+^ and ΔNO signals to suramin suggests that a paracrine mechanism involving ATP release may also contribute to Ca^2+^ wave propagation (as previously reported for bystander radiation damage [45]) and thus also to Ca^2+^−dependent enzymatic NO production. By contrast, the mild inhibition we observed in Ca^2+^−free extracellular medium implies negligible contribution of Ca^2+^ entry to bystander responses [61].

Recent evidence points to a critical involvement of IP_3_R-linked Ca^2+^ signals for the spreading of cytochrome−c induced apoptosis in cellular systems coupled by gap junction channels [64, 65]. Consistent with this tenet, we found that 2-APB, a well known blocker of IP_3_R-dependent calcium release [54] that also inhibits gap junction channels [66], reduced significantly Ca^2+^ wave speed and caused the most pronounced reduction in both ΔCa^2+^ and ΔNO bystander response amplitudes (Figure 5).

The importance of connexins as defense from tumorigenesis and their beneficial role in primary tumors is well supported by several *in vitro* and *in vivo* models [67, 68]. Our results suggest that potentiating bystander effects upregulating connexins by either targeted drug treatments or viral transduction in primary tumors might enhance the therapeutic potential of photodynamic therapy. They also suggest that temporarily inhibiting gap junction communication might reduce unwanted side effects produced by photodynamic treatment of vascular disorders in the retina[11].

## MATERIALS AND METHODS

### Cell culture

1.5–2.0×10^5^ C26GM mouse colon carcinoma cells [29] were plated on 12 mm round glass coverslips and cultured at 37° C, 5% CO_2_ in Dulbecco's modified Eagle's medium (DMEM, Life technologies), supplemented with 2 mM L–glutamine, 10 mM HEPES, 50 μM 2–Mercaptoethanol, 150 U/mL streptomycin, 200 U/mL penicillin and 10% heat–inactivated fetal bovine serum (FBS, Gibco).

### Focal photodynamic injury

AlClPc [30-32] was dissolved in dimethyl sulfoxide at 10 mM concentration and kept in the dark. C26GM cell cultures were incubated with AlClPc (10 μM) and co–loaded with fura–2 AM [36] (15 μM), for 60 min at 37° in DMEM containing pluronic F–127 (0.1%, w/v), and sulphinpyrazone[69] (250 μM). After 60 min of AlClPc and fura–2 incubation, cells were additionally loaded at room temperature for 20 min with CuFl 37,38 at the final concentration of 20 μM. C26GM cells were then transferred to the stage of an upright fluorescence microscope (Bx51, Olympus) and continually superfused with an extracellular medium containing (in Mm): NaCl 150, KCl 5, MgCl_2_ 1, sodium pyruvate 2, Hepes–NaOH 10, D–glucose 5 (pH 7.2, 310 mOsm). When present, CaCl_2_ was added at the final concentration of 1 mM. For focal photodynamic injury, we photo-activated AlClPc for 60 s using a CW 671 nm diode-pumped solid-state laser (Shanghai Dream Lasers) connected to a fluorescence microscope and activated electronically by a transistor-transistor-logic (TTL) command under the control of the image acquisition software. To ensure confined photo-activation within a 5 μm Ø area of a single cell at an irradiance of 60 μW/μm^2^, we launched laser light into a multi-mode step-index fiber optics with 62.5 μm core Ø and projected a sharp demagnified image of the fiber terminal onto the object plane of the microscope using an achromatic collection lens (Thorlabs) and a 650 nm short pass dichroic mirror (Edmund Optics) tilted at 45° and placed right above the objective lens of the microscope.

### Ca^2+^ and NO imaging

The ratiometric Ca^2+^ sensor fura–2 [36] was alternatively excited at 365 nm and 385 nm by light from collimated LEDs (Thorlabs) whereas the turn-on NO sensor CuFL 37,38 was excited by a 470 nm LED (Thorlabs). The three LEDs were activated in rapid sequence for 50 ms each and the activation cycle was repeated once every second. LEDs emissions were filtered through interference band–pass filters centered on the respective peak wavelength, attenuated with a neutral density filter (optical density, 2.12) and conveyed onto the sample by reflection off a DM480HQ dichromatic mirror (Olympus). For both dyes, fluorescence emission was collected through an interference filter (BA495–540HQ, Olympus) using a water immersion objective (40x, N.A. 0.8, LumPlanFL, Olympus). Images were formed on a sCMOS camera (PCO.Edge, 50 ms exposure time/frame) controlled by software developed in the laboratory.

Images were analyzed with software developed in the laboratory using the Matlab platform (Release 14, MathWorks, Inc., Natick, MA, USA). Fura-2 and CuFl traces were generated by averaging pixel signals within regions of interest (ROIs) corresponding to individual cells located at different distance from the irradiated cell. Pseudocolor images were generated using the hue-saturation-value (HSV) visualization algorithm [70]. Hue was used to represent fluorescence changes; value (brightness) carried pixel intensity from a reference image that was either updated on a frame-by-frame basis or obtained as an average over a specified number of frames; saturation was set to 1.0. Frames so constructed were converted to ordinary RGB images by a single call to the Matlab library function hsv2rgb, and displayed.

### Analysis of NO signals

We converted CuFl fluorescence emission *F* at time *t* into NO concentration using the formula

Equation 1 $$[NO](t)\;=\;\frac{1}{K_{ON}}\;\cdot \;\frac{d}{dt}\left(\frac{F(t)}{F_{0}}\right)\;\cdot \;{\left(13.14\;-\;\frac{F(t)}{F_{0}}\right)}^{-1}$$

where k_ON_ is the reaction rate of the CuFl-NO complexation reaction, *F*_0_ is the (constant) pre-stimulus fluorescence and square brackets denote molar concentration. Equation 1 is derived in the Supplementary Methods. To estimate numerically the temporal derivative of the recorded signals, *F*(t)/*F*_0_ data collected after the onset of AlClPc photo-activation were interpolated with a polynomial of order *N* comprised between 8 and 15 using the least square algorithm; the resulting polynomial was then differentiated analytically. Since k_ON_ is unknown[37, 38], all data are presented as percent of the maximal signal computed in the irradiated cell using Equation 1.

### Mathematical model of NO diffusion

The maximal diffusive contribution to NO bystander signals was estimated assuming that NO diffuses freely in the extracellular space as well as across cell membranes. Under these conditions NO concentration is determined by the diffusion equation [71]:

Equation 2 $$\frac{\partial [NO](t)}{\partial t}\;=\;D_{NO}\nabla^{2}[NO](t)$$

[NO](*t*) measured in the irradiated cell using Equation 1 was used as input to the model and Equation 2 was solved numerically in three dimensions using a finite difference approach with a time step Δ*t* = 50 μs and D_NO_ = 3300 μm^2^/s. The domain of Equation 2 and the corresponding boundary conditions are illustrated in Supplementary Figure 3. The domain volume was subdivided in voxels with 10 μm size in the *z* direction and 2 μm size in the *x* and *y* directions. Cells were modelled as having a polygonal base in the *x-y* plane (*z* = 0), reproducing their experimental distribution, and a height *z* = 10 μm. A reflecting boundary at *z* = 0 μM was introduced to describe the effect of the underlying glass, whereas absorbing boundaries were imposed at the horizontal plane *z* = 150 μm and on the vertical planes located at *x* ± 300 μm and *y* ± 300 μm.

### Analysis of Ca^2+^ signals

Ca^2+^ signals were measured as fura-2 emission ratio changes, Δ*R* = *R*(t) − *R*(0), where t is time, *R(t)* is fura-2 emission intensity excited at 365 nm divided by the intensity excited at 385 nm, and *R*(0) indicates pre–stimulus ratio. Estimates of the cytosolic free calcium concentration ([Ca^2+^]_c_) were obtained from ratio values using the Grynkiewicz formula [36]:

Equation 3 $${\left[Ca^{2+}\right]}_{c}(t)\;=\;K_{d}\;\cdot \;\left(\frac{R(t)\;-\;R_{\min}}{R_{\max}\;-\;R(t)}\right)\;\cdot \;\left(\frac{F_{f}}{F_{b}}\right)$$

R_min_ = 0.42 and R_max_ = 13.04 refer to minimum and maximum ratio values recorded in situ with 10 μM ionomycin in the presence of 2 mM EGTA solution and 20mM Ca^2+^ solution, respectively, F_f_/F_b_ = 11.06 is the ratio of the fluorescence values of the Ca^2+^–free and Ca^2+^bound forms at 385 nm and K_d_, the dissociation constant of fura–2 at 22°C, was assumed to be 280 nM.

### Gap junction coupling assay

To visualize gap junction coupling in C26GM cells, we used a novel method based on a combination of patch-clamp and imaging of transmembrane potential [47]. Briefly, glass capillaries for patch clamp recordings were formed on a vertical puller (PP–83, Narishige, Japan) from 1.5-mm outer Ø borosilicate glass (G85150T–4, Warner Instruments) and filled with an intracellular solution containing (in mM): KCl 134, NaCl 4, MgCl_2_ 1, HEPES 20, EGTA 10 (adjusted to pH 7.3 with KOH) and filtered through 0.22-μm pores (Millipore). Pipette resistances were 3–4 MOhm when immersed in the bath. C26GM cells were incubated for 15 min at 37°C in extracellular medium (see above) supplemented with 200 nM of Vf2.1.Cl, a highly sensitive fluorescent sensors of plasma membrane potential [48] kindly provided by Roger Y. Tsien (University of California, San Diego) and pluronic F–127 (0.1% w/v). A cell located near the centre of the field of view was maintained under whole cell voltage clamp conditions using a patch clamp amplifier (EPC-7, HeKa). The patched cell was stimulated by a sinusoidal voltage command (also named carrier wave) delivered to the patch clamp amplifier (frequency 0.5 Hz, amplitude 35 mV). Current and voltage were filtered at 3 kHz by an 8 pole Bessel filter and sampled at 20 kHz using a standard laboratory interface (Digidata 1440A, Molecular Devices) controlled by the PClamp 10 software (Molecular Devices). During electrical stimulation, Vf2.1.Cl fluorescence images were formed using a water immersion objective (60x, 1.0 NA, Fluor, Nikon) and projected on the sCMOS camera. Vf.2.1.Cl fluorescence was excited by light from the 470 nm LED, filtered through a BP460–480 filter (Olympus), attenuated with a neutral density filter (optical density, 2.12) and conveyed onto the sample by reflection off a 515 dcxr dichromatic mirror (Chroma). Fluorescence emission was collected through an ET535/30M filter (Chroma). Vf.2.1.Cl signals elicited by the carrier wave and propagated through the gap junction network were measured as relative changes of fluorescence emission intensity (ΔF/F_0_). At each location, the amplitude of the ΔF/F_0_ signal at the frequency of the carrier wave was extracted using a phase-sensitive detection algorithm (for details, see [47]) and used to quantify the spatial extent of the gap junction network. For these recordings, images were acquired continuously at 10 frames per second with 100 ms exposure time. To synchronize image acquisition and patch clamp recordings, we sampled the 5 V pulse (FVAL) that signals active exposure of the sCMOS camera.

### Apoptosis assay

C26GM cell were co-loaded with AlClPc and fura-2 as described above. After focal photodynamic injury, pSIVA-IANBD and Propidium Iodide (Imgenex) were added directly to the extracellular medium, enriched with CaCl_2_ to a final concentration of 2.5 mM, and cells were imaged by time-lapse microscopy for up to 3 hours. pSIVA-IANBD fluorescence emission was imaged with the same settings used for Vf.2.1.Cl. Propidium Iodide was excited by light from a 535 nm LED attenuated with a neutral density filter (optical density, 2.12) and reflected off a DM560 dichromatic mirror (Olympus), and its fluorescence emission was collected through a long-pass emission filter (590LPV2, Chroma).

### Immunofluorescence and confocal microscopy

C26GM cells, treated or not with AlClPc, were fixed in 4% paraformaldehyde for 20 min at room temperature, rinsed in phosphate buffered saline (PBS), and permeabilized with 0.1% Triton X–100, dissolved in bovine serum albumin (BSA) 1% solution and incubated with anti-cytochrome c antibody (BD Pharmingen). The Alexa Fluor 488-conjugated goat Anti-Mouse IgG (4 μg/ml) was purchased from Life Technologies. Nuclei were counterstained with 1 μg/ml Hoechst 33258 and mounted with ProLong (Life technologies). Images were acquired using a confocal microscope (TCS SP5, Leica) equipped with an oil–immersion objective (63×, 1.25 NA, HCX PL APO, Leica). Laser line intensities and detector gains were carefully adjusted to minimize signal bleed through outside the designated spectral windows.

### qPCR

mRNA was extracted from C26GM cultured cells using RNAeasy kit (Qiagen). cDNA was obtained by reverse transcription of mRNA with random hexamers and ThermoScript RT-PCR system (Life technologies) according to the manufacturer instructions. qPCR was performed on cDNA to amplify Cx26, Cx30, Cx30.3, Cx40, Cx43 and was normalized to GAPDH expression. Amplification was carried out using SYBR Green (Applied Biosystems) on the ABI 7700 sequence detection system equipped with ABI Prism 7700 SDS software (Applied Biosystems) through the following amplification cycles: 50°C: 2 min, 95°C: 10 min, 95°C: 15sec, 60°C: 1 min (40 cycles). For real-time PCR the following primers were used: Cx26f: 5′−CGG AAG TTC ATG AAG GGA GAG AT −3′; Cx26r: 5′−GGT CTT TTG GAC TTT CCT GAG CA −3′; Cx30f: 5′− GTC ATC GGT GGC GTG AAC AAG CAC −3′; Cx30r: 5′− GAG CAG CAT GCA AAT CAC GGA TGC −3′; Cx30.3f: 5′− TCA AAC ATG GGC CCA ATG −3′; Cx30.3r: 5′− GGG AGT CAC AGA GCA AGC −3′; Cx40f: 5′− CTG TCC CCA CCC AGT CAA CT −3′; Cx40r: 5′− CCG TTT GTC ACT ATG GTA GC −3′; Cx43f: 5′− TAC CAC GCC ACC ACC GGC CCA −3′; Cx43r: 5′− GGC ATTTTGGCTGTCGTCAGGGAA −3′; GAPDHf: 5′−ATG TGT CCG TCG TGG ATC TGA C−3′; GAPDHr: 5′−AGA CAA CCT GGT CCT CAG TGT AG−3′.

Quantification of connexin mRNA expression relative to GAPDH was performed using the ΔΔCT method.

### Data analysis and statistics

Unless otherwise stated, statistical comparisons of means for paired samples were made by one-way heteroscedastic Student t-test. p-values are indicated by letter *p* and *p* < 0.05 was selected as the criterion for statistical significance. In figures, asterisks were used as follows: * *p* ≤ 0.05; ** *p* ≤ 0.01; *** *p* ≤ 0.001.

## SUPPLEMENTARY MATERIAL AND FIGURES



## Abbreviations

2-APB: 2 Aminoethoxydiphenyl borate
AlPCc: Aluminum Phthalocyanine Chloride
Ca2+: Calcium ion
CaM: Calmodulin
CBX: Carbenoxolone
CuFL: {4,5-Bis[(6-(2-ethoxy-2-oxoethoxy)-2-methylquinolin-8 ylamino) methyl] 6-hydoxy-3-oxo-3H-xanthen-9-yl}benzoic acid FL
CPA: Cyclopiazonic Acid
Cx: Connexin
Cyt C: Cytochrome c
EGTA: Ethylene Glycol-bis(beta-aminoethyl ether)-N,N,N',N'-Tetraacetic Acid
ER: Endoplasmatic Reticulum
FFA: Flufenamic Acid
GM-CSF: Granulocyte Macrophage - Colony Stimulating Factor
IP3: Inositol 1,4,5-trisphosphate
IP3R: IP3 receptors
L-NAME: L-Nω-nitroL-Arginine Methyl Ester
L-NIO: N-iminoethyl-L-ornithine
NO: Nitric Oxide
NOS: Nitric Oxide Synthase
PI: Propidium Iodide
PMCA: Plasma membrane Ca2+ATPase
PSIVA: Polarity Sensitive Indicator of Viability and Apoptosis
RNS: Reactive Nitrogen Species
ROS: Reactive Oxygen Species
ROI: Region Of Interest
SERCA: Sarco/Endoplasmatic reticulum Ca^2+^ATPase
