# Supplementary material for: Critical role of gap junction communication, calcium and nitric oxide signaling in bystander responses to focal photodynamic injury
Source: Oncotarget. 2015 Mar 12;6(12):10161–74. doi: 10.18632/oncotarget.3553 (PMC4496347; doi:10.18632/oncotarget.3553)
Supplement: Supplementary file 1 [file oncotarget-06-10161-s001.pdf]

Critical role of gap junction communication, calcium and nitric oxide signaling in bystander responses to focal photodynamic injury

Supplementary Material

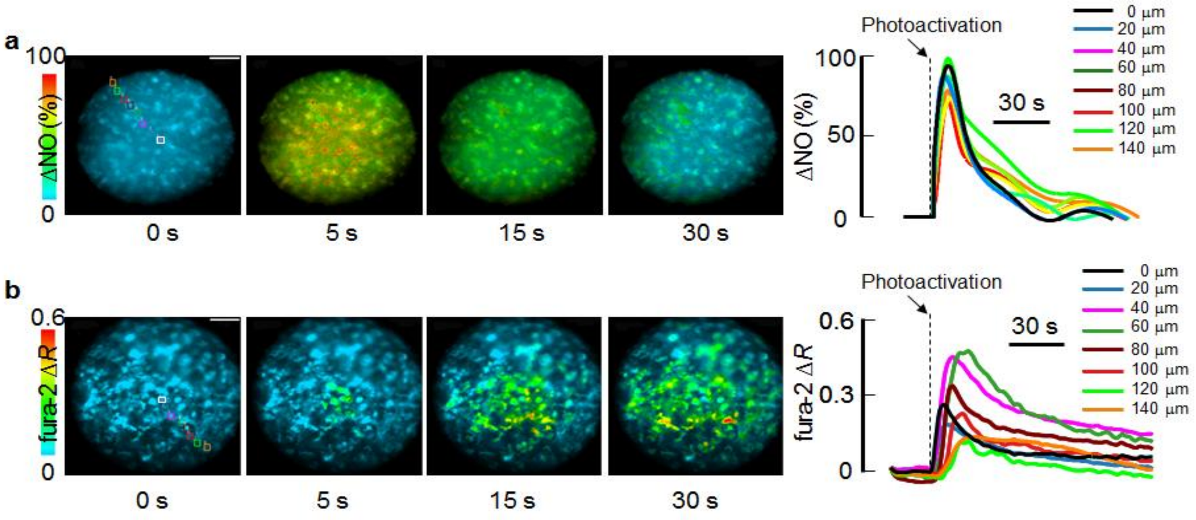

Supplementary Figure 1. *In vivo* focal photodynamic injury. NO and Ca^{2+} signals triggered by focal photodynamic injury were monitored by intravital microscopy in C26GM tumors grown for 5 days in a dorsal skinfold chamber applied to Balb/c mice. Shown are representative false−color images of cytosolic NO (**a**) and Ca^{2+} (**b**) concentration changes (Δ); the irradiated cell is encased in a white region of interest (ROI); scale bar, 50 μm. Single-cell fluorescence traces were obtained as pixel averages from the corresponding (color-matched) ROIs; irradiated cell responses are shown as black traces.

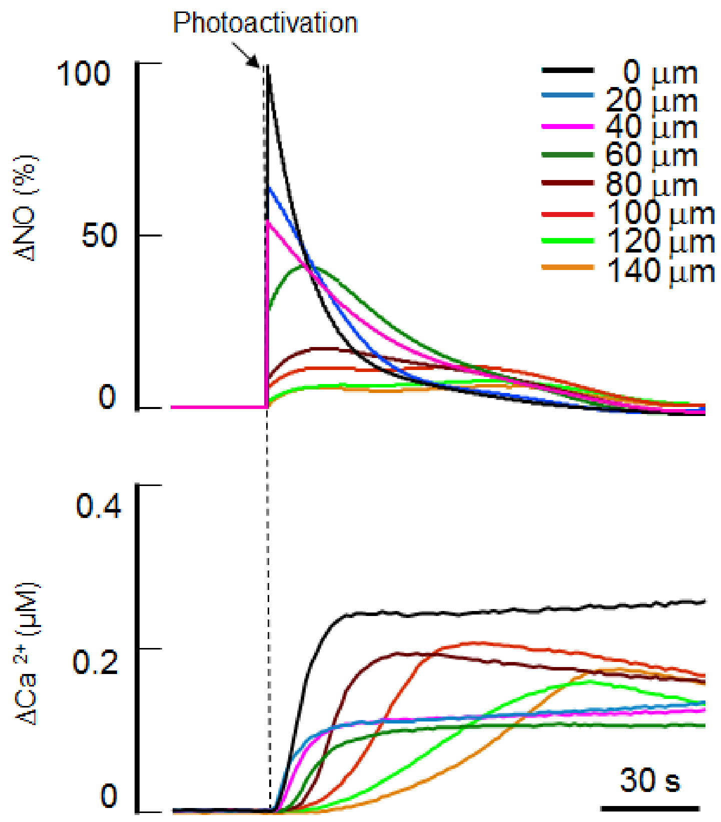

Supplementary Figure 2. Focal photodynamic injury, i.e. photo–activation of the photosensitizer AlClPc for 60 s in a single cell of a MCA-203 mouse fibrosarcoma cell culture. Single-cell fluorescence traces obtained as pixel averages from the corresponding ROIs; irradiated cell responses are shown as black traces; the vertical dashed line marks the onset of laser irradiation; ΔNO data were normalized to the corresponding maximal response in the irradiated cell (see Methods);

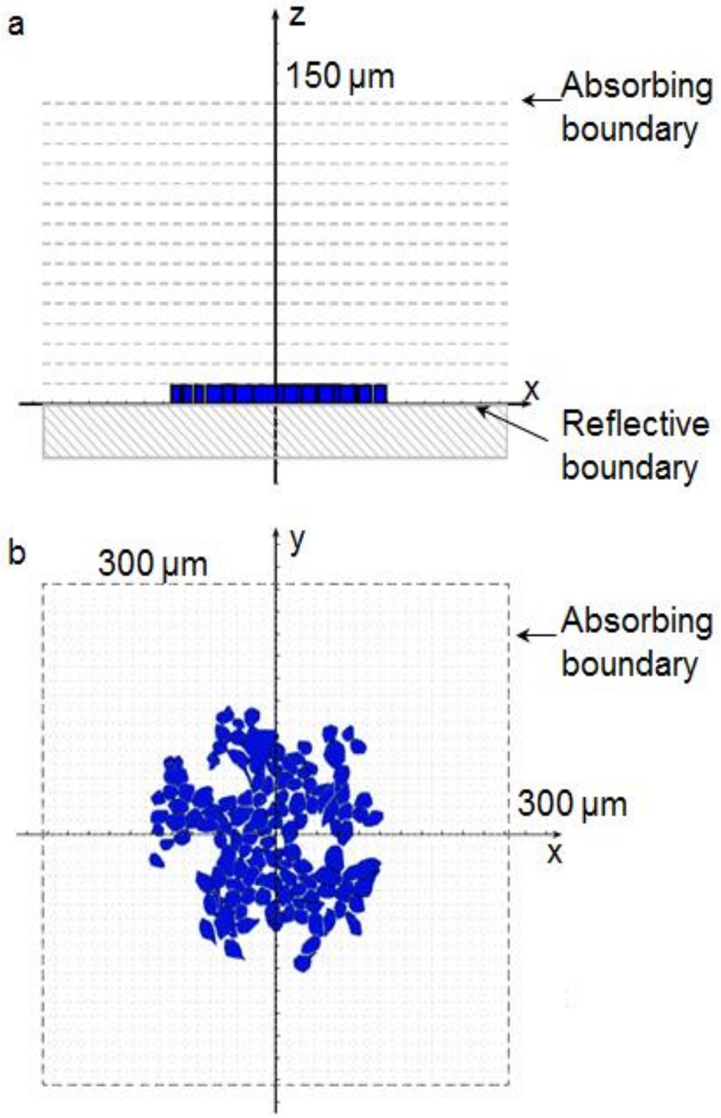

Supplementary Figure 3. Scheme of mathematical model geometry and boundaries.

SUPPLEMENTARY METHODS

Intravital microscopy. Adult male BALB/c (27-30 grams) mice were purchased from Charles River Laboratories and maintained under pathogen-free conditions in the animal facilities of the Venetian Institute of Molecular Medicine. Experiments were performed according to state guidelines and approved by the local ethics committee (Università degli Studi di Padova, Comitato Etico di Ateneo per la Sperimentazione Animale – CEASA, Project n. 79 BIS / 2010). Animals were anesthetized with an i.p. dose of 80mg/kg Ketamine and 5mg/kg Xylazine and maintained at 37° C on a steel heating plate. Using a laminar flow hood to maintain a sterile field, dorsal skinfold window chambers were surgically implanted using the procedure described in [39]. After surgery, mice were treated twice a day for three days with Tramadol i.m. at a dose of 10mg/kg/die and checked daily for general health and chamber-skin interaction. Three days after surgery, 0.5×10^6 C26GM colon carcinoma cells were labelled with 5 μM CFDA-SE (Life technologies) and dropped within the chamber in a total volume of 20 μl. At day 5-7 of tumor growth, the tumor bearing mouse was anesthetized with ketamine/xylazine (80mg/kg and 5mg/kg) and prepared for focal photodynamic injury under conditions of intravital microscopy. The tumor mass within the dorsal chamber was incubated with 20 μM AlClPc and co-loaded for 60 min with fura-2 AM (30 μM) by micro-injection with a glass micropipette with an opening ∅ of 10 μm connected to pneumatic pico-pump (SYS-PV820, WPI) delivering 10 psi of pressure for 10 s. The incubation medium contained also pluronic F–127 (0.01% w/v), and sulphinpyrazone (250μM) to prevent dye sequestration and secretion. For NO detection, after one hour AlClPc incubation, the tumor was loaded with CuFl at the final concentration of 40 μM by micro-injection with a glass micropipette as described above. LED emission was attenuated with neutral density filters (O.D. 2.12 for fura-2 and 2.81 for CuFL) and images were acquired

using 200 ms exposure time/frame. Due to spectral overlap with CuFL, CFDA-SE fluorescence emission was measured before CuFl loading and subtracted offline before image processing. All other imaging parameters and methods were identical to those used for *in vitro* experiments.

Derivation of Equation 1

The mechanism responsible for NO sensing by CuFl is a two-step process based (1) on the coordination of a fluorophore ligand Fl to a metal ion Cu^{II}

$$\mathrm{Cu}^{\mathrm{II}} + \mathrm{Fl} \longleftrightarrow \mathrm{Cu}^{\mathrm{II}}\mathrm{Fl}$$

which quenches dye fluorescence; (2) NO reduction of the metal center and concomitant release of the nitrosated ligand restores fluorescence emission. The latter is described by the irreversible reaction [38]

$$\mathrm{Cu}^{\mathrm{II}}\mathrm{Fl} + \mathrm{NO} \longrightarrow \mathrm{Cu}^{\mathrm{I}} + \mathrm{NOFl}$$

which corresponds to the differential equation

$$\frac{d[\mathrm{NOFl}](t)}{dt} = k_{\mathrm{ON}}[\mathrm{NO}](t)[\mathrm{CuFl}](t) \qquad \text{Equation S1}$$

where k_{ON} is the (forward) reaction rate and square brackets denote molar concentrations of the reactants. For any $t_0 < t$, mass conservation imposes that

$$[\mathrm{CuFl}](t) = [\mathrm{CuFl}](t_0) - [\mathrm{NOFl}](t) \qquad \text{Equation S2}$$

Combining Equation S1 and Equation S2 yields

$$\frac{d[\mathrm{NOFl}](t)}{dt} = k_{\mathrm{ON}}[\mathrm{NO}](t)\left\{[\mathrm{CuFl}](t_0) - [\mathrm{NOFl}](t)\right\} \qquad \text{Equation S3}$$

In our setup, the maximal fluorescence emission under saturating NO levels corresponded to 14.14 fold increase. Therefore the total fluorescence emission F at any time t can be expressed as

$$F(t) = \alpha\{[\mathrm{CuFl}](t) + 14.14\ [\mathrm{NOFl}](t)\}$$ Equation S4

where α is an unknown proportionality constant which lumps all factors depending on the experimental setup. Let us define F_0 as the (constant) pre–stimulus fluorescence emission at time $t_0 < t$. Assuming that only a negligible fraction of the available CuFl dye has undergone complexation with NO under pre–stimulus resting condition, we can express F_0 as

$$F_0 \cong \alpha[\mathrm{CuFl}](t_0)$$ Equation S5

Taking into account Equation S4 and Equation S5, the normalized fluorescence emission is given by

$$\frac{F(t)}{F_0} = 1 + 13.14 \frac{[\mathrm{NOFl}](t)}{[\mathrm{CuFl}](t_0)}$$ Equation S6

which does not depend on the unknown parameter α. Taking the time derivative of Equation S6 yields

$$\frac{d}{dt}\left(\frac{F(t)}{F_0}\right) = \frac{13.14}{[\mathrm{CuFl}](t_0)} \cdot \frac{d[\mathrm{NOFl}](t)}{dt}$$ Equation S7

Combining Equation 7 and Equation 3 shows that

$$\frac{d}{dt}\left(\frac{F(t)}{F_0}\right) = 13.14\ k_{\mathrm{ON}}[\mathrm{NO}](t)\left(1 - \frac{[\mathrm{NOFl}](t)}{[\mathrm{CuFl}](t_0)}\right)$$ Equation S8

Finally, combining Equation S8 and Equation S6 and solving for $[\mathrm{NO}](t)$ yields the formula

$$[\mathrm{NO}](t) = \frac{1}{k_{\mathrm{ON}}} \cdot \frac{d}{dt}\left(\frac{F(t)}{F_0}\right) \cdot \left(13.14 - \frac{F(t)}{F_0}\right)^{-1}$$

which is listed as Equation 1 in the main text.
